# Efficacy of Tislelizumab in Lung Cancer Treatment: A Systematic Review and Meta-Analysis of Randomized Controlled Trials

**DOI:** 10.7759/cureus.80609

**Published:** 2025-03-15

**Authors:** Waqas Ul Bassar, Oboseh J Ogedegbe, Asfia Qammar, FNU Sumia, Mujahed Ul Islam, Sandipkumar S Chaudhari, Olanipekun L Ntukidem, Areeba Khan

**Affiliations:** 1 Internal Medicine, Ayub Medical Teaching Institution, Abbottabad, PAK; 2 Internal Medicine, Lifeway Medical Center Limited, Abuja, NGA; 3 Pathology, Dow Medical College, Southlake, USA; 4 Pathology, Peoples University of Medical and Health Sciences for Women, Nawab shah, PAK; 5 Infectious Diseases, University Hospital of North Midlands, Stoke-on-Trent, GBR; 6 Cardiothoracic Surgery, University of Alabama at Birmingham, Birmingham, USA; 7 Family Medicine, University of North Dakota School of Medicine and Health Sciences, Fargo, USA; 8 Internal Medicine, Trinity Health, Ann Arbor, USA; 9 Critical Care Medicine, United Medical and Dental College, Karachi, PAK

**Keywords:** immunotherapy, lung cancer, meta-analysis, pd-1 inhibitors, tislelizumab

## Abstract

This systematic review and meta-analysis evaluated the efficacy of tislelizumab, alone or in combination with chemotherapy, in patients with lung cancer. A comprehensive literature search was conducted across PubMed, Embase, Web of Science, and CENTRAL databases until February 15, 2025. Only randomized controlled trials (RCTs) comparing tislelizumab with control treatments in lung cancer patients were included. The primary outcomes assessed were overall survival (OS) and progression-free survival (PFS). Four phase-III RCTs involving 1,837 patients were included in the analysis. The results demonstrated that tislelizumab significantly improved OS (hazard ratios (HR): 0.72, 95% CI: 0.63-0.81) and PFS (HR: 0.61, 95% CI: 0.54-0.68) compared to control treatments. Subgroup analyses revealed consistent benefits across both non-small-cell lung cancer (NSCLC) and small-cell lung cancer (SCLC) populations, with no significant differences between cancer types. Similarly, the efficacy of tislelizumab was comparable whether administered as monotherapy or in combination with chemotherapy. Low heterogeneity was observed among the included studies, suggesting consistency in treatment effects. Follow-up duration across studies ranged from 14.2 to 16.7 months. These findings indicate that tislelizumab, either alone or combined with chemotherapy, is an effective treatment option for lung cancer patients, demonstrating significant improvements in survival outcomes. However, further high-quality RCTs are needed to validate these results, particularly in SCLC patients, where evidence is limited to a single study. Future research should also consider patient-specific factors such as age, gender, and comorbidities to refine treatment strategies.

## Introduction and background

About 85% of all cases of lung cancer are non-small-cell lung cancer (NSCLC), with small-cell lung cancer (SCLC) making up the remaining 15%. Lung cancer is still the largest cause of cancer-related mortality globally [1-2]. Despite advancements in diagnosis and treatment, the prognosis for lung cancer patients remains poor, particularly in advanced-stage disease [3].

Over the last decade, immune checkpoint inhibitors (ICIs) have revolutionized lung cancer treatment by enhancing anti-tumor immune responses [4]. Among these, tislelizumab, a PD-1 monoclonal antibody designed to reduce Fcγ receptor binding, thereby limiting antibody-dependent phagocytosis of T cells, has shown promising efficacy in both NSCLC and SCLC [5-6].

While several clinical trials have demonstrated the efficacy of tislelizumab in NSCLC and ES-SCLC, findings have been inconsistent across different patient populations and treatment regimens [7-10]. Previous reviews have examined individual PD-1/PD-L1 inhibitors, but a comprehensive analysis specifically comparing tislelizumab's efficacy and safety across lung cancer subtypes is lacking [11]. Additionally, existing studies have reported varied outcomes regarding the relationship between PD-L1 expression levels and response to tislelizumab, creating uncertainty for clinical decision-making.

Despite tislelizumab’s increasing adoption in clinical practice, no systematic evaluation has comprehensively assessed its comparative effectiveness in different lung cancer settings or established clear predictive biomarkers for patient selection. This systematic review and meta-analysis aim to address these research gaps by analyzing survival outcomes (OS, PFS) associated with tislelizumab across different lung cancer subtypes, treatment combinations, and biomarker profiles. By synthesizing the available evidence, this study will provide clinicians with evidence-based insights for optimizing tislelizumab-based treatment strategies in lung cancer management.

## Review

Methodology

Literature Search

From the beginning to February 15, 2025, a thorough literature search was carried out in PubMed, Embase, Web of Science, and the Cochrane Central Register of Controlled Trials (CENTRAL). The search approach comprised Medical Subject Headings (MeSH) phrases and pertinent keywords associated with "PD-1 inhibitors," "Tislelizumab," "small-cell lung cancer (SCLC)," "non-small-cell lung cancer (NSCLC)," "chemotherapy," and "randomised controlled trials (RCTs)." The search was narrowed down using Boolean operators (AND/OR). To find more acceptable papers, the reference lists of pertinent studies and systematic reviews were manually filtered. There were no linguistic limitations. Two authors conducted the search. Discussion was used to settle any disputes.

Study Selection

Two independent reviewers screened the titles and abstracts of all retrieved studies. Full-text articles of potentially eligible studies were assessed based on predefined inclusion and exclusion criteria. Only randomized controlled trials (RCTs) evaluating the efficacy and safety of tislelizumab alone or in combination with chemotherapy in patients with NSCLC or SCLC were included. Studies that were non-randomized, observational, preclinical, reviews, editorials, or case reports were excluded. Studies without a control arm were also not included. Disagreements between reviewers were resolved through discussion or consultation with a third reviewer.

Quality Assessment

The Cochrane risk of bias 2 (RoB 2) instrument, which assesses bias across five domains-randomization procedure, deviations from intended treatments, missing outcome data, assessment of outcomes, and selection of reported results, was used to evaluate the risk of bias in the included RCTs. Every study was categorized as having a high risk of bias, some issues, or low risk. The quality assessment was completed independently by two reviewers, and any disagreements were discussed or discussed with a third reviewer.

Data Extraction

Data from the included studies were extracted using a spreadsheet developed on Microsoft Excel. One author extracted data and the second author cross-checked it and entered it into RevMan for data analysis. Data extracted from included studies were author name, year of publication, trial phase, trial name, groups, sample size, type of lung cancer, and whether tislelizumab was given alone or in combination with other therapies and outcomes. Outcomes assessed in this study included overall survival (OS) and progression-free survival (PFS). Any disagreement between the two authors was resolved through discussion.

Statistical Plan

To conduct all statistical analyses, Review Manager (RevMan) software was used. Using the inverse variance method, the pooled effect sizes were presented as hazard ratios (HRs) with 95% CIs for time-to-event outcomes, such as OS and PFS. The I2 statistic and the Chi-square test (Cochran’s Q) were used to evaluate the heterogeneity among the included studies. Low heterogeneity was defined as an I2 value of less than 25%, moderate heterogeneity as 26-50%, and high heterogeneity as >50%. A random-effects model was employed if significant heterogeneity (I2 >50%) was noted; if not, a fixed-effects model was utilized.

To explore potential sources of variation in treatment effects, subgroup analyses were conducted based on lung cancer type (NSCLC vs. SCLC) and intervention type (Tislelizumab monotherapy vs. Tislelizumab plus chemotherapy). Differences between subgroups were assessed using interaction tests, and a p-value <0.05 was considered statistically significant for subgroup differences.

Results

**Figure 1:**
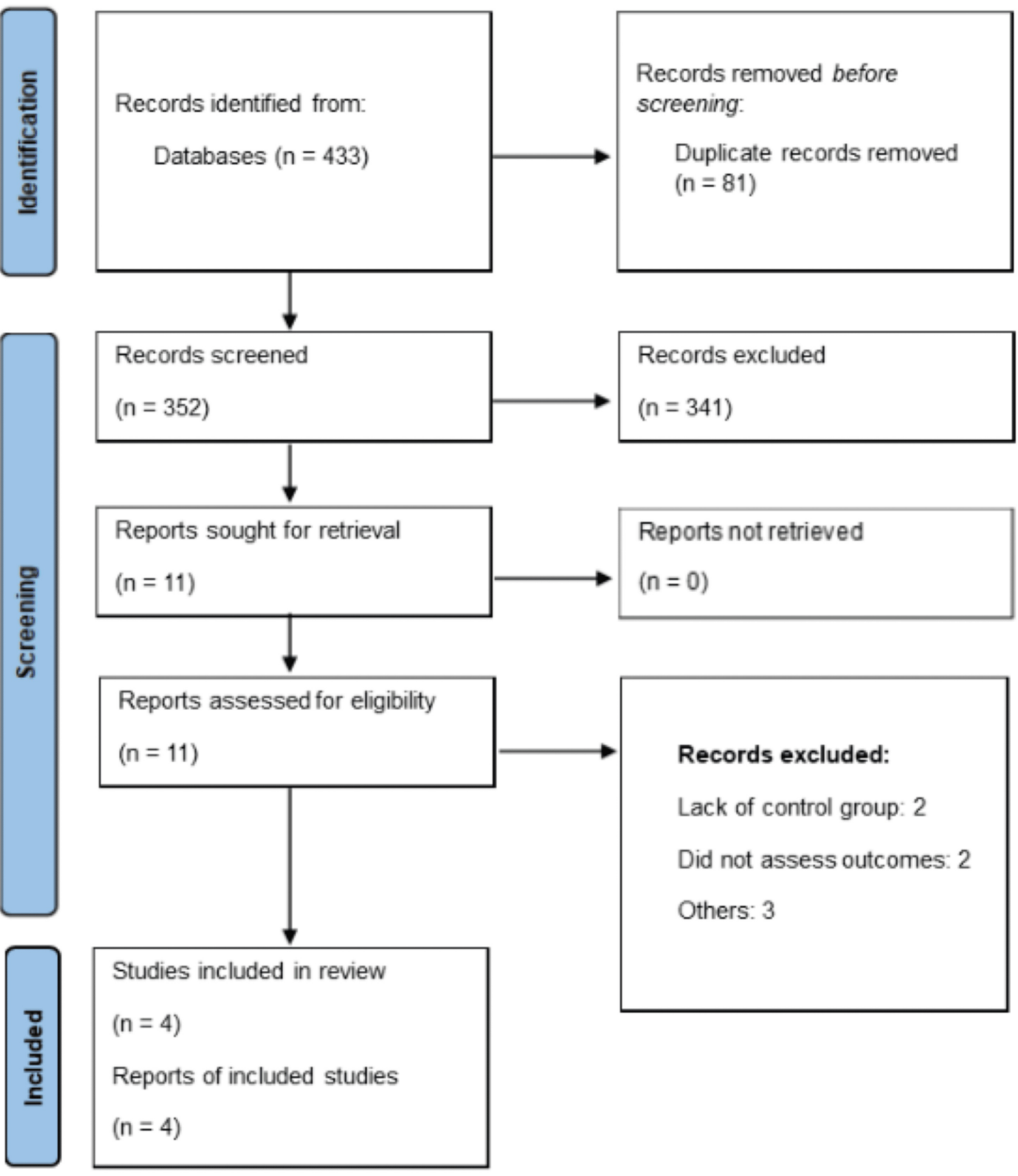
PRISMA flowchart of study selection The image was created by the authors of this article.

**Figure 2:**
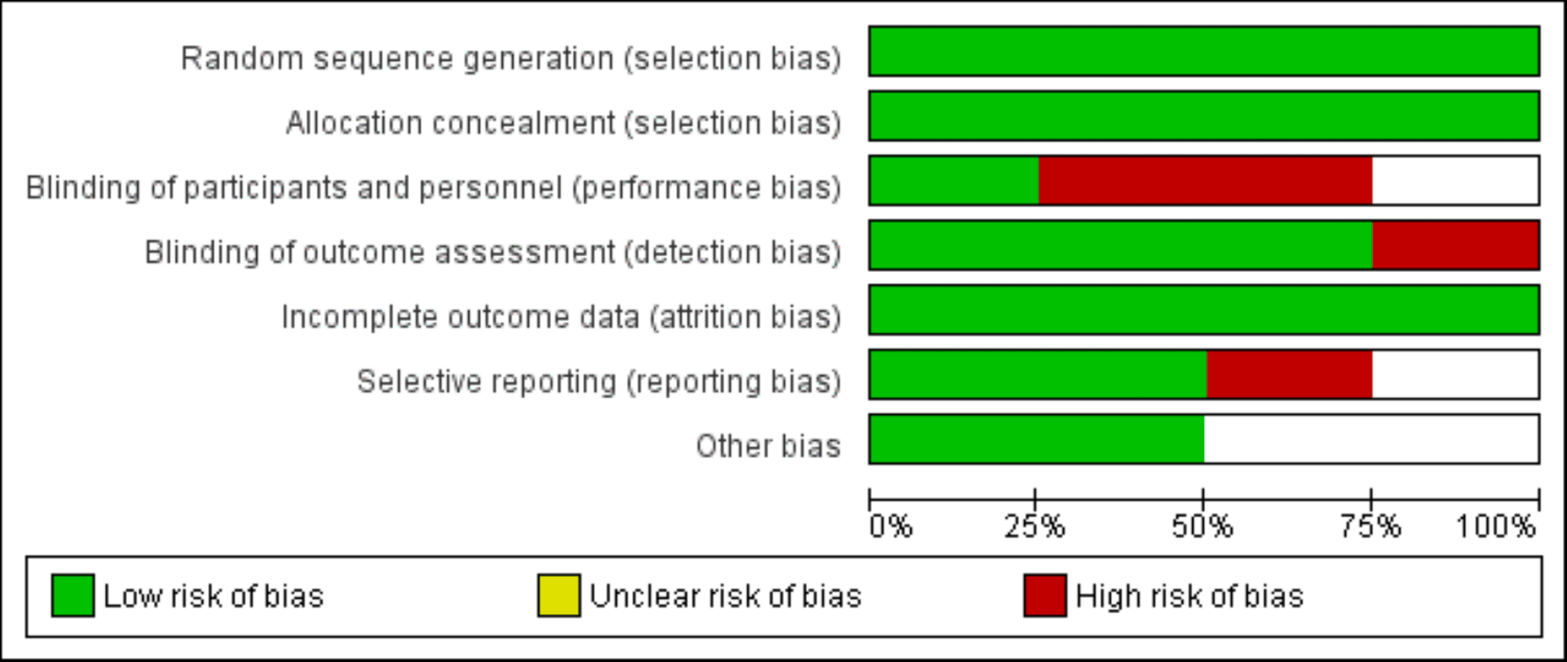
Risk of bias assessment of included studies The image was created by the authors of this article.

Figure 1 shows the PRISMA flowchart of study selection. Through online database searching, we found 433 studies. After removing duplicate studies, we assessed 352 studies using their title and abstracts. Full-texts of 11 studies were obtained and detailed assessment was done. Finally, four RCTs were included in this meta-analysis encompassing 1837 subjects. Table 1 presents the characteristics of the included studies. All included studies were phase-III RCTs. Follow-up duration of included studies ranged from 14.2 to 16.7 months. Three studies included subjects with NSCLC. In three of the included studies, tislelizumab was given in combination with chemotherapy, while only one study gave tislelizumab alone. Figure 2 presents the quality assessment of the included studies.

**Table 1:** Characteristics of included studies

Author	Phase	Trial Name	Groups	Sample Size	Lung Cancer Type	Follow-up	Age	Male (n)
Cheng et al. 2024 [12]	Phase III	RATIONALE-312	Tislelizumab	227	Small	14.2 months	63	186
			Control	230			62	186
Lu et al. 2024 [13]	Phase III	RATIONALE-304	Tislelizumab	223	Non-small	16.1 months	63	186
			Control	111			62	186
Wang et al. 2024 [14]	Phase III	RATIONALE-307	Tislelizumab	120	Non-small	16.7 months	60	107
			Control	121			62	111
Zhou et al. 2023 [15]	Phase III	RATIONALE-303	Tislelizumab	535	Non-small	16 months	61	416
			Control	270			61	206

Overall Survival (OS)

**Figure 3:**
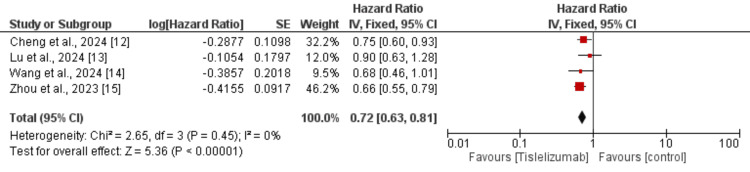
Effect of tislelizumab on overall survival References [12-15] The image was created by the authors of this article.

Four studies were used in the pooled analysis to assess the effect of tislelizumab on OS in patients with lung cancer. As shown in Figure 3, OS is significantly greater in patients receiving tislelizumab compared to the control group (HR: 0.72, 95% CI: 0.63 to 0.81). No heterogeneity was reported among the study results.

**Figure 4:**
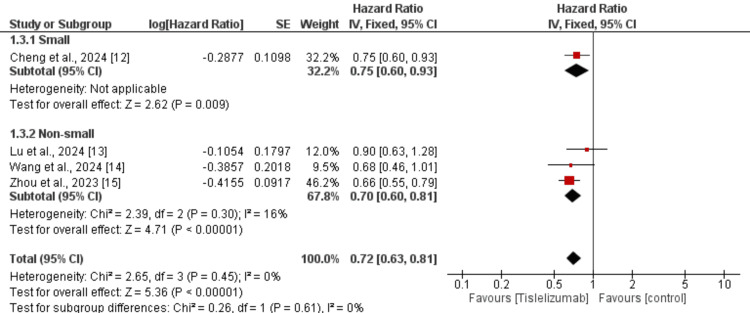
Subgroup analysis on effect of tislelizumab on overall survival (based on lung cancer type) References [12-15] The image was created by the authors of this article.

**Figure 5:**
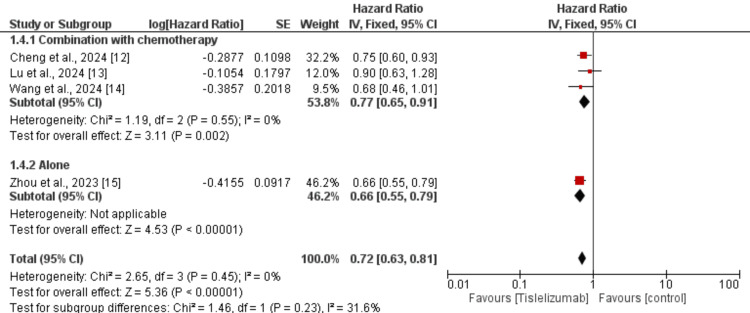
Subgroup analysis on effect of tislelizumab on overall survival (based on tislelizumab) References [12-15] The image was created by the authors of this article.

We performed subgroup analysis based on the type of lung cancer whether it is NSCLC or SCLC and the results are presented in Figure 4. In both subgroups, OS was greater in subjects receiving tislelizumab compared to the control groups and the difference in the effect of tislelizumab was not significantly different between the two groups (p-value>0.05). We also performed subgroup analysis based on whether tislelizumab was given alone or in combination with chemotherapy and the results are presented in Figure 5. In both subgroups, OS was greater in subjects receiving tislelizumab compared to the control groups and the difference in the effect of tislelizumab was not significantly different between the two groups (p-value>0.05).

Progression-Free Survival (PFS)

**Figure 6:**
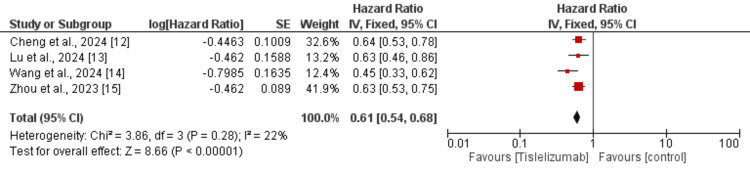
Effect of tislelizumab on progression-free survival References [12-15] The image was created by the authors of this article.

Four studies were used in the pooled analysis to assess the effect of tislelizumab on PFS in patients with lung cancer. As shown in Figure 6, OS is significantly greater in patients receiving tislelizumab compared to the control group (HR: 0.61, 95% CI: 0.54 to 0.68). Low heterogeneity was reported among the study results (I-Square: 22%).

**Figure 7:**
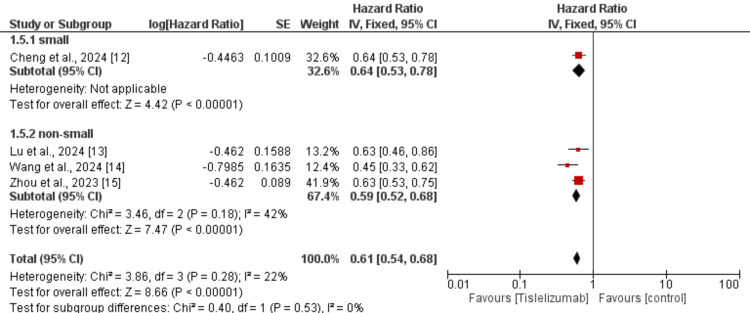
Subgroup analysis on effect of tislelizumab on progression-free survival (based on lung cancer type) References [12-15] The image was created by the authors of this article.

**Figure 8:**
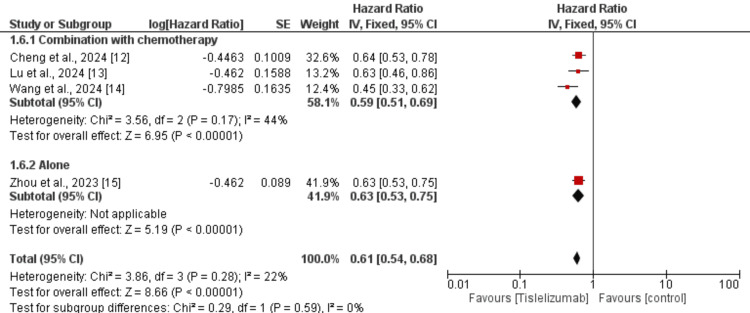
Subgroup analysis on effect of tislelizumab on progression-free survival (based on tislelizumab) References [12-15] The image was created by the authors of this article.

We performed subgroup analysis based on the type of lung cancer whether it is NSCLC or SCLC and the results are presented in Figure 7. In both subgroups, PFS was greater in subjects receiving tislelizumab compared to the control groups and the difference in the effect of tislelizumab was not significantly different between the two groups (p-value>0.05). We also performed subgroup analysis based on whether tislelizumab was given alone or in combination with chemotherapy and the results are presented in Figure 8. In both subgroups, PFS was greater in subjects receiving tislelizumab compared to the control groups and the difference in the effect of tislelizumab was not significantly different between the two groups (p-value>0.05).

Discussion

This meta-analysis of four phase-III RCTs demonstrated that tislelizumab, either alone or in combination with chemotherapy, significantly improved OS and PFS in patients with lung cancer compared to the control group. The pooled HRs indicated a 28% reduction in the risk of death (HR: 0.72, 95% CI: 0.63-0.81) and a 39% reduction in the risk of disease progression (HR: 0.61, 95% CI: 0.54-0.68) with tislelizumab. Subgroup analyses revealed that these survival benefits were consistent across both NSCLC and SCLC subgroups, with no significant differences in treatment effect based on cancer type. Similarly, the efficacy of tislelizumab was comparable regardless of whether it was administered as monotherapy or in combination with chemotherapy. These findings reinforce the role of tislelizumab as a promising therapeutic option for lung cancer, further supporting its integration into standard treatment regimens. The low heterogeneity observed in the pooled analyses suggests a high level of consistency across studies, strengthening the reliability of these results.

Tislelizumab, a monoclonal immunoglobulin G4 antibody targeting PD-1, is an immunotherapeutic agent approved in China for the treatment of classical Hodgkin lymphoma (cHL) [16]. It has also demonstrated efficacy in managing various solid malignancies, including esophageal squamous cell carcinoma (ESCC) [15], gastric and gastroesophageal junction adenocarcinoma [17], as well as lung cancer. Its safety profile in lung cancer patients has been evaluated through several phase 1 clinical trials [7, 18]. Additionally, findings from phase 2 clinical trials have indicated that tislelizumab, when administered in combination with chemotherapy, is an effective treatment strategy for advanced lung cancer [7].

Tislelizumab is a monoclonal antibody that targets the programmed death-1 (PD-1) receptor, a key immune checkpoint that tumors exploit to evade immune system attack [7]. By blocking PD-1, tislelizumab restores T-cell activation and proliferation, enhancing anti-tumor immune responses. This leads to increased cytotoxic activity against cancer cells, ultimately improving survival outcomes. Unlike some other PD-1 inhibitors, tislelizumab has been engineered to minimize binding to Fcγ receptors, which may reduce the risk of antibody-dependent cellular cytotoxicity of T cells and improve therapeutic efficacy [19-20]. When combined with chemotherapy, tislelizumab may further enhance anti-tumor effects by inducing immunogenic cell death, which increases tumor antigen presentation and strengthens immune recognition [21]. This dual approach of immune checkpoint blockade and chemotherapy creates a synergistic effect, contributing to the observed improvements in OS and PFS.

The findings of this meta-analysis highlight the potential role of tislelizumab as a valuable treatment option for lung cancer, either as monotherapy or in combination with chemotherapy. Given its significant improvement in OS and PFS, tislelizumab could be considered as a first-line or second-line therapy for patients with NSCLC and extensive-stage small-cell lung cancer (ES-SCLC). The comparable efficacy between monotherapy and combination therapy suggests that treatment selection could be tailored based on patient characteristics and disease burden.

This meta-analysis has several limitations. Firstly, it included only four RCTs, which may restrict the generalizability of the findings. Secondly, while three studies evaluated PFS and OS in NSCLC, only one study focused on SCLC, highlighting the need for more robust evidence in this subgroup. Additionally, due to limited data availability, we were unable to conduct subgroup analyses based on factors such as age, gender, and comorbidities. Despite these limitations, our findings support tislelizumab as an effective and well-tolerated treatment, either alone or with chemotherapy. Further, high-quality RCTs are essential to validate these results and refine treatment strategies.

## Conclusions

This meta-analysis of four phase-III RCTs demonstrated that tislelizumab, whether as monotherapy or in combination with chemotherapy, significantly improves OS and PFS in patients with lung cancer. The consistent survival benefits across both NSCLC and SCLC suggest its broad therapeutic potential. The comparable efficacy between monotherapy and combination therapy highlights its versatility in clinical practice. Despite some limitations, including a small number of included studies and a lack of subgroup analyses based on patient characteristics, these findings support tislelizumab as a promising treatment option. Further, well-designed RCTs are needed to validate these results and optimize treatment strategies, ensuring personalized and evidence-based care for lung cancer patients.
